# Targeted therapies in colorectal cancer—an integrative view by PPPM

**DOI:** 10.1186/1878-5085-4-3

**Published:** 2013-01-28

**Authors:** Suzanne Hagan, Maria C M Orr, Brendan Doyle

**Affiliations:** 1 Department of Life Sciences Glasgow, Caledonian University, Glasgow, G4 0BA, UK; 2 Personalised Healthcare and Biomarkers, AstraZeneca, Alderley Park, Macclesfield, Cheshire, SK10 4TG, UK; 3 Department of Histopathology, Trinity College, St. James's Hospital, Dublin, 8, Ireland

**Keywords:** Clinical decision-making process, Molecularly targeted treatment, Therapy response, Prediction, Therapy monitoring, Anti-EGFR, Anti-VEGF, Monoclonal antibody therapy, CRC management, Integrative medical approach

## Abstract

In developed countries, colorectal cancer (CRC) is the third most common malignancy, but it is the second most frequent cause of cancer-related death. Clinicians are still faced with numerous challenges in the treatment of this disease, and future approaches which target the molecular features of the disorder will be critical for success in this disease setting. Genetic analyses of many solid tumours have shown that up to 100 protein-encoding genes are mutated. Within CRC, numerous genetic alterations have been identified in a number of pathways. Therefore, understanding the molecular pathology of CRC may present information on potential routes for treatment and may also provide valuable prognostic information. This will be particularly pertinent for molecularly targeted treatments, such as anti-vascular endothelial growth factor therapies and anti-epidermal growth factor receptor (EGFR) monoclonal antibody therapy. *KRAS* and *BRAF* mutations have been shown to predict response to anti-EGFR therapy. As EGFR can also signal via the phosphatidylinositol 3-kinase (*PI3K*) kinase pathway, there is considerable interest in the potential roles of members of this pathway (such as *PI3K* and PTEN) in predicting treatment response. Therefore, a combined approach of new techniques that allow identification of these biomarkers alongside interdisciplinary approaches to the treatment of advanced CRC will aid in the treatment decision-making process and may also serve to guide future therapeutic approaches.

## Review

### Introduction to colorectal cancer

Colorectal cancer (CRC) is the fourth leading cause of cancer-related mortality worldwide, accounting for over 600,000 deaths annually [1]. Estimates for CRC in the 40 European countries studied in 2008 [1] indicated that CRC was the most common cancer (436,000 cases, 13.6% of total) and the second most common cause of death from cancer (212,000 deaths, 12.3% of total). Recent genome-wide analyses of solid tumours, including CRC, have shown mutations in between 20 and 100 protein-encoding genes [2-7]. A number of key genetic and epigenetic alterations which lead to malignant transformation have been identified in CRC, and these include aberrations in genes involved in the chromosomal instability (CIS) pathway, the microsatellite instability (MSI) pathway, the hMYH pathway and the CpG island methylation pathway [8]. Recent data have shown that *KRAS* and *BRAF* mutations predict response to anti-epidermal growth factor receptor (EGFR) therapy reviewed in [9]. Great strides have been made in the early detection and diagnosis of CRC, including population-based screening, which has the potential to prevent up to 60% of CRC deaths reviewed in [10]. Despite this, however, up to 56% of newly diagnosed CRC patients present with either nodal or distant metastases [11]. Prognosis is poor for these patients, with an overall 5-year survival rate of 6.6%–11.9% for Dukes D patients [11,12]. Therefore, further developments are essential in order to increase the 5-year survival rate and to improve the overall quality of life (QoL) for patients with this disease.

### The role of predictive, preventive and personalised medicine in CRC

Predictive, preventive and personalised medicine (PPPM) endeavours to promote a paradigm shift in our current healthcare approach. The PPPM approach aims to predict individual predisposition before onset of the disease, to provide targeted preventive measures and to create personalised treatment algorithms tailored to the individual. The concept aims to move from delayed intervention to predictive medicine tailored to the person, from reactive to preventive medicine and from disease to wellness. It is hoped that this will provide a more cost-effective management of major diseases, such as cancer, in the future. The critical role of PPPM in the modernisation of healthcare systems has been acknowledged as a priority by global and regional organisations and health-related institutions, such as the Organisation of United Nations, the European Union and The National Institutes of Health. In CRC, the potential value of biomarkers for PPPM is strong, and there have been recent increased efforts to incorporate the use of such markers into healthcare systems. Biomarkers could be used to:

• Identify disease predisposition

• Identify early disease and aid appropriate timely treatment intervention

• Aid molecular classification of the disease, with a view to provide better disease understanding and more effective, targeted treatment options

• Identify patient populations that are more likely to derive clinical benefit from current and future treatment options.

Whilst the identification of biomarkers of predisposition and of early stage disease is critical, the focus of this review article will be on the use of markers as an aid to the classification of CRC and their role as potential companion diagnostics.

### Classification of CRC

Classification of CRC has traditionally been based on histopathological features. Molecular studies have allowed a significant appreciation of the heterogeneous nature of CRC. However, it has long been known, based on morphological criteria, that CRC is not a homogenous disease. For example, even before the advent of molecular classification of tumours, it was noted that the rare, but histologically distinct ‘medullary carcinoma’ occurs almost exclusively on the right side of the colon and is associated with an improved prognosis compared to the standard histological types [13].

More recently, the molecular changes underlying these phenotypical appearances have been elucidated. To use the example above, it has now been shown that medullary carcinoma of the colon shows MSI with loss of the DNA mismatch repair (MMR) enzymes, such as MLH-1, MSH-2, MSH-6 and PMS-2 [13]. It is also now known that this molecular signature is shared by the more common serrated tumour pathway. This loss of DNA mismatch repair activity may be caused by mutation of one of these genes, as seen in hereditary non-polyposis colorectal cancer (HNPCC). More commonly, these tumours arise sporadically, and the loss of MMR function is due to epigenetic silencing of one of the genes, as a result of promoter methylation. High-level MSI (MSI-H) comprises 15% of sporadic CRC, and these are positively correlated with patients being female, over 60 years of age, having *BRAF* mutations and being right-sided tumours [14]. It is interesting to note that although there is a continuous increase in the rate of MSI-H tumours as one progresses proximally from the rectum to the ascending colon, the previously popular view of dichotomous (proximal and distal) tumours has been challenged by recent data [15].

In addition to the finding that CpG island methylator phenotype (CIMP)-high tumours have particular phenotypical and prognostic features, they also evolve from a different precursor lesion than those tumours which are microsatellite stable (MSS) and show CIS. CIMP-high tumours form part of the serrated pathway and develop from precursor lesions, which have a different histological appearance to standard colonic adenomas [16,17]. In addition to the known difference in prognosis for these tumours, there has also been the suggestion that they may also be associated with a lack of clinical benefit from standard 5-fluorouracil (5-FU)-based chemotherapy [18,19].

**Figure 1:**
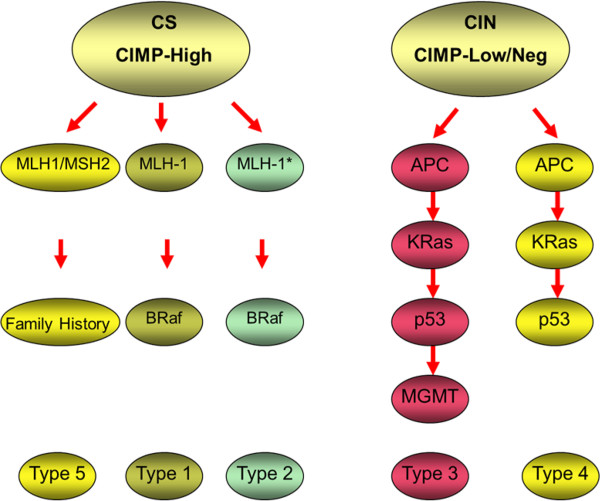
**Molecular classification of CRC as described by Jass **[17]**.** Tumours are divided primarily on the basis of CIMP status and microsatellite stability. Group 1 tumours show methylation of MLH1 and B-Raf mutations. They are characterised by CIMP+ and MSI-H, arise from serrated polyps and account for approximately 12% of CRC. Group 2 tumours are similar, but show only partial methylation of MSH1 associated with B-Raf mutation. They are CIMP+, MSS/microsatellite instable-low (MSI-L), arise from serrated polyps and account for approximately 8% of CRC. Group 3 tumours show not only mutations in APC, Kras and/or p53 but also methylation of MGMT. These tumours are CIMP-L and MSS/MSI-L, and show CIS. They can arise in either serrated or classical adenomas, and account for approximately 20% of CRC. Group 4 tumours are the classical type described in Vogelstein's original model [20], which show mutations in APC, Kras and/or p53. These tumours demonstrate CIS and are CIMP- and MSS. They arise in classical adenomas and make up approximately 57% of CRC. Group 5 tumours are those tumours arising in the familial cancer syndrome, HNPCC. They show mutations in one of the DNA MMR genes, are CIMP-, but MSI-H and account for approximately 3% of CRC (adapted from Ibrahim and Arends, [146]).

This molecular sub-typing of CRC has advanced to the stage where we can now begin to consider a molecular classification-based approach for CRC. Jass suggested one such classification and proposed five molecular sub-classifications (see Figure 1), based on levels of MSI and CIMP [17]. 

### Introduction to current therapies

Four key approaches are currently used for the treatment of CRC: surgery, chemotherapy, radiotherapy and targeted therapies. The mainstay of CRC treatment is surgery. In early stage disease (stage 0 or I), surgical excision can be used without need for further treatment options, as the recurrence rate for node-negative T1 colorectal cancer is very low [21]. Many studies have now shown that adjuvant therapy has a survival benefit for patients with stage III disease, and therefore, this is the standard of care. The situation is not yet clear for patients with stage II CRC, however, in which there is somewhat conflicting evidence regarding the benefit of adjuvant therapy. It is agreed that ‘high-risk’ stage II patients should be offered adjuvant therapy, as they are the most likely to derive a benefit, although there is currently some debate regarding the exact definition of ‘high-risk’ stage II CRC. Patients with stage IV disease require chemotherapy or targeted therapies combined with surgery, where appropriate. Whilst surgery, chemotherapy and radiation therapy are key contributors to CRC treatment, the remainder of this article will focus on targeted therapies.

### Targeted therapies in the treatment of CRC

As a consequence of improved understanding of the molecular pathology of cancer, a number of targeted agents have been developed which have demonstrated improved outcome in metastatic CRC (mCRC) patients, with combination chemotherapy. Among the first of these drugs to be developed and approved for use by the Food and Drug Administration (FDA) in mCRC were the following:

1. Bevacizumab (Avastin™, Genentech/Roche, CA, USA), a monoclonal antibody targeted to vascular endothelial growth factor (VEGF) [22]; and

2. The anti-EGFR monoclonal antibodies cetuximab (Erbitux™, Imclone Systems, NJ, USA [23]) and panitumumab (Vectibix™, Amgen, CA, USA [24]).

#### Anti-angiogenic drugs (bevacizumab)

Folkman et al. [25] first suggested the concept of targeting angiogenesis in cancer over 40 years ago. In 2004, the FDA approved bevacizumab as a first-line treatment for patients with mCRC. This followed a phase III study in 2004 on mCRC patients by Hurwitz et al. [22], which demonstrated that the inclusion of this drug to 5-FU combination therapy resulted in a ‘statistically significant and clinically meaningful improvement in survival’ among mCRC patients. Bevacizumab is a recombinant humanised monoclonal antibody that specifically targets VEGF-A, which is synthesised during tumour growth. Bevacizumab is thus defined as an anti-angiogenic drug due to its ability to prevent VEGF from interacting with appropriate receptors in vascular endothelial cells. As a result, cell signalling pathways that enhance angiogenesis, and thus the blood supply for tumours, are diminished. Bevacizumab is commonly used in combination with standard chemotherapeutic agents (e.g. 5-FU) as a first-line treatment for patients with mCRC and improves the overall survival (OS) of these patients by approximately 5 months [26,27]. In theory, by specific targeting of endothelial cells, anti-angiogenic agents (such as bevacizumab) may avoid potential tumour cell resistance, thus indicating their usefulness in treating metastatic disease. Additionally, in contrast to EGFR-based therapies, bevacizumab is usually well tolerated by patients, and skin-based side effects are relatively rare [28]. This will be discussed in more detail later.

##### Limitations and side effects of bevacizumab

By targeting angiogenesis, a generalised biological phenomenon in the human body, bevacizumab-based therapies may result in toxicities affecting multiple organs. For example, in the Hurwitz et al. trial, a significantly higher rate of adverse effects was noted, including hypertension, anorexia and proteinuria [22]. A recent retrospective analysis of 228 Japanese patients with mCRC [29] showed that 96 (42%) did not receive bevacizumab as part of their treatment regime. In the majority of cases (76%), this was due to bevacizumab-specific contraindications, whilst an Italian study [30] demonstrated that 10% of mCRC patients had serious treatment-related toxicities. Of some concern is the correlation between gastrointestinal perforation, which occurs in 1%–2% of bevacizumab-treated patients, and mortality rates. This was reviewed recently in a community-based cohort of 1,953 CRC patients [31], and the authors found that most toxic events were successfully addressed by surgery. Other researchers have noted the limited survival benefits observed in some bevacizumab studies, whereby addition of bevacizumab to oxaliplatin-based chemotherapy significantly improved progression-free survival (PFS); however, OS differences did not reach statistical significance nor was response rate improved [32], whilst some preclinical and clinical studies have reported that VEGF inhibition actually increased tumour invasiveness and metastasis, suggesting that bevacizumab may indirectly stimulate new blood vessel growth (and tumour dissemination) through a VEGF-independent mechanism [33,34].

Although considered rare, some cutaneous side effects of bevacizumab therapy have been observed, including exfoliative dermatitis, peripheral sensory neuropathy, skin discolouration and dryness [35]. However, it should also be noted that two studies by researchers at Yale University have suggested a potential association between skin rash occurrence in bevacizumab-treated mCRC patients and a *positive* drug response [36,37], whilst Manzoni et al. [38] have suggested a role for the increased presence of circulating endothelial cells (CEC) as future predictive biomarkers for bevacizumab-treated mCRC patients.

##### Biomarkers and response to bevacizumab—potential predictive markers?

There is evidence that indicates that biomarkers may be of value in determining response to bevacizumab, as mentioned above. Such markers include vascular imaging, hypertension and polymorphisms affecting components of the VEGF pathway. In addition, profiles of circulating cytokines, growth factors and angiogenesis-related molecules may also prove valuable as prognostic and/or predictive markers.

##### Use of imaging tools

Imaging approaches, such as dynamic contrast enhanced (DCE)-magnetic resonance imaging (MRI) and ^18^F-fluorothymidine (FLT)-positron emission tomography (PET), may be used to assess the anti-angiogenic activity of bevacizumab. Small studies undertaken in advanced biliary tract cancer [39,40] and breast cancer [41] have indicated that such approaches are promising as potential predictors of response. In the malignant glioma study, FLT-PET at 1–2 and 6 weeks was found to be a positive predictor of a survival benefit, whilst in biliary tract cancers, changes in FLT-PET (after 2 cycles of bevacizumab) were found to be a significant predictor of PFS and OS. In breast cancer patients receiving neoadjuvant chemotherapy plus bevacizumab, DCE-MRI indicated that greater decreases in angiogenic volume were associated with patients who derived a clinical benefit [42]. Whilst data in the CRC setting is more limited, there have been some indications that DCE-MRI may predict tumour shrinkage in response to combined bevacizumab and cytotoxic chemotherapy in CRC liver metastases [43]. However, further assessments using larger cohorts are necessary to determine if this approach has future value as a predictive biomarker of response to treatment.

##### Hypertension as a potential biomarker

Recent studies have indicated that the development of bevacizumab-induced arterial hypertension may serve as a potential predictive biomarker. Clinically, patients treated with bevacizumab have shown a rapid rise in blood pressure, and 5–18% have experienced grade 3 or 4 hypertension [44]. Hypertension, therefore, may be a useful biomarker of VEGF activity and predict the anti-angiogenic activity of bevacizumab. A number of small, single-arm studies have evaluated this in pancreatic cancer [45], renal carcinoma [46] and in CRC [47]. These studies have indicated that bevacizumab-induced hypertension (or the necessity for increased antihypertensive medication during bevacizumab treatment) was associated with extended PFS or OS. Further analysis carried out retrospectively on phase III trials in metastatic breast cancer and non-squamous, non-small cell lung cancer provides support for the biomarker as a predictive marker of clinical benefit [48,49]. In CRC, however, the situation is less clear. Hurwitz et al. [50] undertook a retrospective analysis of two CRC studies and found that in only one of these studies did hypertensive changes (during bevacizumab therapy) predict a clinical benefit, as measured by PFS and OS, whilst Dewdney et al. [51] undertook a phase II study of bevacizumab-induced hypertension of 45 patients with poor-risk colorectal liver-only metastases. Although 15% of patients developed ≥ grade 1 hypertension (whilst receiving neoadjuvantx chemotherapy), and 4% developed grade 3 hypertension, no correlation was found between this condition and radiological response rate, PFS or OS.

This uncertainty could indicate that the predictive value of bevacizumab-induced hypertension might not extend to all cancers or treatment regimens, and thus, further work on CRC in large scale, prospective studies is required to elucidate the role of this as a potential biomarker.

##### Circulating biomarkers

Baseline measurements of circulating VEGF levels have been shown to be prognostic in a number of tumour types, including mCRC, lung cancer and renal-cell cancer. These studies also indicated that circulating VEGF levels were not predictive of treatment response to bevacizumab-based treatment regimes [52]. A recent study undertaken by Duda et al. [53] looked at the concentrations of VEGF, placental growth factor (PlGF), soluble VEGF receptor 1 (sVEGFR-1) and sVEGFR-2. These were measured in plasma and urine at baseline and during treatment in patients with locally advanced rectal cancer. In this study, they found that pre-treatment plasma sVEGFR-1, an endogenous blocker of VEGF and PlGF, and a factor linked with ‘vascular normalisation’ were associated with both primary tumour regression and the development of adverse events after neoadjuvant bevacizumab and chemoradiation [53].

A number of studies have been undertaken in an attempt to identify other circulating prognostic or predictive markers of bevacizumab therapy. A review of these studies can be found in Jubb and Harris [54] and by Wilson et al. [55]. In a study of 32 mCRC patients, Abajo et al. [56] utilised ELISAs and multiplex bead assays to determine if serum cytokines were predictive of bevacizumab efficacy. This group noted that high baseline serum levels of EGF and macrophage-derived chemokine and low levels of interleukin-6 (IL-6), IL-8 and IL-10 were correlated with likelihood of improved response. As mentioned previously, work by Manzoni et al. [38] on a group of 24 mCRC patients (undergoing bevacizumab treatment) indicated increased CECs and the apoptotic fraction of CECs as future (mutually independent) predictive biomarkers for this mCRC population, and appear to confirm other similar studies on CECs [57-61].

##### Polymorphisms in the VEGF pathway

Interest in the role of genetic variants in the VEGF pathway, as biomarkers for bevacizumab treatment outcome, has increased in light of the finding that heritability accounts for almost 80% of the variance seen in VEGF levels [62]. In metastatic breast cancer, Schneider et al. [48] identified two VEGF genotypes (VEGF-2578AA and VEGF-1154AA) which were significantly associated with improved OS in the bevacizumab plus paclitaxel treatment group. However, the same polymorphisms did not show any association with a PFS benefit. Both of these polymorphisms have also been associated with OS in a retrospective study of patients with mCRC receiving either FOLFIRI (leucovorin, fluorouracil, and irinotecan) plus bevacizumab, or XELIRI (capecitabine and irinotecan) plus bevacizumab [63]. A study undertaken by Lambrechts et al. [64] identified a locus in VEGFR1, which correlates with increased VEGFR1 expression and poor outcome of bevacizumab-treated patients with metastatic pancreatic adenocarcinoma and metastatic renal-cell carcinoma, although no work has as yet been undertaken in the CRC setting.

Despite the observations regarding the above polymorphisms, no data exist to indicate the biological significance of these variants and their role in response to treatment. As a result, additional work would provide a better understanding of their relevance.

#### Anti-EGFR therapy

EGFR is a 170 kD glycoprotein located in chromosome 7 [65] and is a member of the transmembrane tyrosine kinase receptor family, ErbB. EGFR is known to be over-expressed in tumours of epithelial origin, including CRC. EGFR activation is triggered by binding of peptide growth factors (of the EGF family) to the extracellular domain of the receptor and subsequently initiates both the RAS/RAF/MAPK and the phosphatidylinositol 3-kinase (*PI3K* signalling pathways. As a result, EGFR is directly involved in cell proliferation and survival, and therefore contributes to metastatic progression. Two classes of anti-EGFR therapies exist. The first group is composed of monoclonal antibodies to EGFR, whilst the second class includes tyrosine kinase inhibitors (TKIs), such as erlotinib and gefitinib.

##### Tyrosine kinase inhibitors

TKIs serve to block the cascade of reactions crucial to tumour development and survival; therefore, their anticancer properties have become an important focus for drug development by the pharmaceutical industry. To date, TKIs have obtained FDA approval for use in a range of cancers, including chronic myeloid leukaemia, acute lymphocytic leukaemia, non-small cell lung cancer and pancreatic cancer. Moreover, the use of the TKIs sunitinib, sorafenib and pazopanib into clinical care has doubled the PFS of patients with renal-cell cancer [66,67]. As a result, sorafenib (originally developed as a BRAF inhibitor) is currently being tested in phase II trials for mCRC. Whilst current phase III trials of novel targeted agents for mCRC (specifically the small molecule TKIs, erlotinib and gefitinib) have been approved for targeting several cancers, neither has yet been approved for treating CRC. Additionally, the TKI imatinib has been used to treat advanced gastrointestinal stromal tumours; however, these tumours represent a very small proportion (<3%) of GI malignancies, are thus classed in a different category to CRC and are termed as connective tissue tumours.

Recent work has indicated the ability of TKIs to strongly suppress PI3K signalling (and possibly other pro-survival pathways) in CRC, suggesting that these treatments may have great therapeutic value [68]. Despite this, however, there has been no FDA approval granted to TKIs as new targeted agents for the treatment of mCRC since 2004. As a consequence, we will confine our discussions of anti-EGFR therapies to monoclonal antibodies.

##### Monoclonal antibodies to EGFR

Two EGFR antagonists, cetuximab (Erbitux™, Imclone Systems) and panitumumab (Vectibix™, Amgen) were FDA-approved for the treatment of mCRC in 2004 and 2006, respectively. Cetuximab is a chimeric immunoglobulin IgG1 monoclonal antibody that targets the ligand-binding domain of EGFR, whilst panitumumab is a human IgG2 monoclonal antibody. Both antibodies prevent EGFR autophosphorylation by binding to the extracellular domain and thus inhibiting activation of the downstream cell signalling pathways, MAPK and *PI3K*. Each antibody has been approved for the treatment of advanced CRC based on various parameters, including QoL, PFS and OS when used individually or in combination with standard chemotherapeutics.

##### Cetuximab

An early phase II trial that assessed the efficacy of cetuximab [69] by treatment-refractory CRC patients demonstrated the drug to have ‘modest activity’ that was well tolerated by patients. This study also noted that presence and severity of drug-induced acneiform skin rash correlated positively with survival [69]. Whilst in a seminal study of 329 mCRC patients, Cunningham et al. [70] showed that cetuximab had ‘clinically significant activity’ in patients with irinotecan-refractory CRC when given alone or in combination with this drug. Combination therapy patients demonstrated a higher response rate, longer median progression times and longer median survival times. Similarly, in a phase II trial of cetuximab by Vincenzi et al. [71], this anti-EGFR therapy was found to be clinically active in mCRC patients who were refractory to other regimens (irinotecan and oxaliplatin). In 2006, a multicentre study by Lenz et al. [72] demonstrated that cetuximab was well tolerated by mCRC patients who were previously unresponsive to irinotecan, oxaliplatin and fluoropyrimidines. As in previous studies, skin rash severity was correlated with drug response. Jonker et al. [73] undertook a study of cetuximab in treatment-resistant mCRC patients and noted that this drug therapy improved OS, PFS and preserved QoL measures, whilst a rash of grade 2 or above was robustly associated with improved survival.

##### Panitumumab

Following on from the successful use of cetuximab, a second anti-EGFR antibody, panitumumab, was introduced. Similar to cetuximab, panitumumab demonstrated significant anti-tumour activity in treatment-refractory mCRC patients, as well as a good safety profile. Studies of panitumumab by Gibson et al. [74] in a randomised phase III trial also demonstrated a reduction in tumour progression (46%) of patients with mCRC. Improved OS was positively correlated with severity of an acneiform skin rash, which was observed in 90% of subjects undergoing this treatment regime. Further, in a randomised phase III trial comparing panitumumab with other treatment regimens, including bevacizumab, Wainberg and Hecht [75] concluded that this therapy had ‘significant activity’ when used alone and improved PFS when compared with best supportive care (BSC). This finding was also observed in a study on treatment-refractory mCRC patients by Van Cutsem et al. [76]. Moreover, response rates of 10% were observed in panitumumab-treated subjects versus none for BSC. Yet, it should be noted that in this study, there was no change in OS. In a randomised phase III trial, Siena et al. [77] showed improved PFS in panitumumab-treated mCRC patients who were previously unresponsive to other regimens. Therefore, as a consequence of its success in addressing mCRC, panitumumab was approved by the FDA for use in the treatment of CRC and lung cancer.

##### Limitations and side effects of EGFR therapies

Skin toxicities are a common adverse event associated with EGFR inhibitors, with up to 80% of patients presenting with a skin rash (acneiform) on the scalp, face, neck and upper trunk [78]. This rash tends to occur within 3 weeks after therapy initiation, and it has been demonstrated that this response is reversible upon therapy cessation. In general, however, most studies suggest that EGFR therapies are well tolerated by patients. Both cetuximab and panitumumab have shown acceptable safety profiles in patients with mCRC. For example, Mitchell et al. [79] recently demonstrated that panitumumab (combined with irinotecan) had an acceptable toxicity profile in mCRC patients. Although in some cases, hypersensitivity reactions have been observed with cetuximab treatment [80,81]. By contrast, hypersensitivity reactions are rare with panitumumab which has been indicated as an alternative treatment for patients at risk from cetuximab-induced anaphylaxis.

##### Biomarkers and response to anti-EGFR therapy—potential predictive markers?

Skin toxicity as a biomarker of response to anti-EGFR therapy

Research has suggested that adverse skin events following anti-EGFR therapies may be indicative of patient response. For example, cetuximab-induced skin rash in mCRC patients has been strongly correlated with improved PFS, OS and response rates in a number of studies [69,82,83]. Additionally, panitumumab has demonstrated a strong association between skin toxicities and improved outcome [84,85]. Further pre-emptive [86] and prospective toxicity studies will enable researchers to clarify the mechanisms underlying the skin rash response in these therapies.

Major progress has been made using anti-EGFR therapies, including the understanding of biomarkers and their contribution to drug response. Despite the success of anti-EGFR therapy, however, not all patients obtain the same benefits. This has led to the search for biomarkers which might indicate the patient population that would benefit most from anti-EGFR therapy. As such therapies are expensive and potentially toxic, the search for such biomarkers is driven, not only from the clinical and scientific community, but also from the payor community. Therefore, specific targeting of patients who would benefit from this treatment is being actively pursued.

EGFR polymorphisms/mutations and overexpression

To date, a small number of pharmacogenetic studies have identified EGFR polymorphisms as potential biomarkers in predicting response to anti-EGFR therapies in mCRC and non-small cell lung cancer [87-89]. By contrast to lung adenocarcinoma, however, EGFR mutations in CRC are somewhat rare [90-92], thus limiting their utility as biomarkers at present.

Immunohistochemical (IHC) studies of CRC tumours indicated that patients with EGFR-positive tumours benefit from anti-EGFR therapy [93]. Since 2004, the combination of a diagnostic test for EGFR (EGFR PharmDx™ Kit, Dako, Cambridgeshire, UK), and the subsequent application of cetuximab or panitumumab in EGFR-positive colon carcinomas has been approved by FDA, reviewed in [94]. However, subsequent studies have shown that EGFR-negative tumours may also benefit from cetuximab therapy [69,95,96]. These studies appear to indicate that analysis of EGFR expression via IHC may not be as reliable in predicting the efficacy of EGFR therapy. As a consequence of these disappointing results, researchers have instead undertaken a search for alternative biomarkers. These investigations have lead to a focus on downstream effectors of EGFR signalling, including RAS family members, BRAF and the *PI3K* pathway, amongst others. Each of these potential biomarkers will be discussed individually. 

**Table 1:** **A number of approved*KRAS*mutation tests available for use in CRC**

**Test name**	**Manufacturer**	**Test details**
Cobas *KRAS* Mutation Test	Roche Diagnostics	The test detects mutations in codons 12, 13 and 61 of the *KRAS* gene
AmoyDx *KRAS* test	Amoy	Detects the seven most common activating mutations of the *KRAS* gene in cancer tissue
SURVEYOR Scan K-RAS Mutation Detection Kit	Transgenomic Inc	Detects mutations in exon 2 of the K-RAS gene (codon 12 and 13)
PyroMark Q24 KRAS Assay-Kit	Qiagen	Able to detect all major and minor known mutations in the KRAS codons 12, 13 and 61, and, in addition, allows the discovery of new mutations as well
TheraScreen: K-RAS Mutation Kit	Qiagen	K-RAS Kit will detect seven K-RAS mutations in codons 12 and 13 of the K-RAS oncogene

1. *KRAS*

*KRAS* is a member of the RAS proto-oncogene family which is transiently activated by the action of ligand binding of EGFR. The RAS family also includes *NRAS* and *HRAS*. *KRAS* mutations have been reported in approximately 40% of human CRC [97-100]. The majority (85%) of these mutations occur in codons 12 and 13, with a smaller number occurring in codons 61, 117 and 146 [101-103]. Oncogenic mutation in *KRAS* renders the protein constitutively active, thus maintaining it in the GTP-bound conformation.

Due to the common occurrence of *KRAS* mutations in CRC, a number of studies have examined their potential clinical relevance. Some studies have shown that the presence of a *KRAS* mutation may have prognostic significance, as reviewed in [92]. This effect appears to be borderline and confined to certain mutations (particularly the G12V mutation). Moreover, the effect may be confined to certain stages of CRC.

The importance of *KRAS* mutation was truly realised with the advent of anti-EGFR monoclonal antibodies as a treatment for mCRC. These were introduced into clinical use around 2004 and showed reasonable response rates of 10%–15% in mCRC. However, their role was revolutionised by the discovery that patients whose tumours harbour mutant forms of *KRAS* do not respond to anti-EGFR therapy [104]. Although the results of this study were statistically significant, patient numbers were small and there was a need for greater sample sizes. Subsequent studies with larger numbers confirmed these findings [85,105]. Meta-analyses of the available randomised controlled trials have gone on to further support these results, demonstrating that a benefit from anti-EGFR therapy is only seen in patients whose tumour is wild-type for *KRAS*[106-108]. In these studies, no difference was found in the predictive effects of mutations in different codons.

Based on these and other studies, the American Society of Clinical Oncology recommended that patients who are candidates for anti-EGFR therapy should have their tumour tested for *KRAS* mutation. If a codon 12 or 13 mutation is detected, these patients should not be treated with anti-EGFR therapy [109]. Similarly, the Canadian Expert Group has stated that *KRAS* status should be determined whenever anti-EGFR therapy is being considered in the setting of mCRC [110,111]. In 2009, the FDA updated the product labels for cetuximab and panitumumab, indicating that patients with CRC tumours harbouring *KRAS* mutations were unlikely to derive benefit from these therapies. However, these guidelines may have to be re-visited, as it has been shown (in one study) that patients with a G13D mutation showed improved survival compared to patients with other mutations, indicating that this patient group may respond to therapy [112]. This is, therefore, an area that will require further study.

2. KRAS mutation heterogeneity

Although there is now widespread agreement that the presence of a *KRAS* mutation indicates that a patient is unlikely to respond to anti-EGFR therapy, there is some concern around the area of tumour heterogeneity. Some studies have shown a strong correlation in mutation status between primary tumours and distant metastases [113,114]. Yet, other studies have suggested that there is significant heterogeneity in the expression of *KRAS* mutations within a tumour [115-118] and also between primary tumour samples and lymph node metastases [113,117]. This could theoretically lead to false negative results, i.e. there is a *KRAS* mutation present within a tumour, but the portion sampled for testing does not contain the mutation (or there are insufficient numbers of tumour cells with the mutation) to enable detection. Whilst this issue has been raised in the literature, to date, no reliable data have been provided to suggest that appropriate sampling protocols are in place. A robust sampling method would address this concern [118].

3. Approach used for KRAS mutation testing

The analysis of tumour samples for *KRAS* mutations is undertaken using samples of fresh-frozen or paraffin-embedded tumour material. A pathology review of the material is essential to establish that the sample contains sufficient tumour cells for analysis. If necessary, enrichment for tumour material may be carried out using tumour microdissection. DNA is then extracted from the sample and used for molecular testing using sequencing or pyrosequencing-based approaches, or an allele-specific PCR-based approach. Currently, there are no FDA-approved protocols for *KRAS* testing. However, a number of laboratory-based assays can be utilised, provided they are run under CAP certification or other CMS-approved certification (as required by the CLIA regulations of 1988). In countries where CE marking is accepted, there are a number of approved *KRAS* mutation tests available for use in CRC. A summary of these tests is listed in Table 1 below.

4. The cost/benefit ratio of KRAS testing

The use of mutation testing has obvious benefits to patients by preventing exposure of those patients unlikely to respond, to potential toxic effects of a given drug regime. Moreover, there are significant economic benefits. Studies have demonstrated that the investment in testing for *KRAS* and *BRAF* (discussed later) results in significant cost savings [119,120]. For example, as early as 2009, researchers were estimating that routine testing for *KRAS* mutations in CRC patients would save approximately $740 million a year, whilst more recently, Vijayaraghavan et al. [121] have estimated that *KRAS* mutation testing saves $7,500–$12,400 per patient in the USA and €3,900–€9,600 per patient in Germany.

##### Other potential biomarkers of mCRC

The finding of a link between *KRAS* mutation status and anti-EGFR therapy has led to the investigation of other downstream effectors as potential biomarkers. The most investigated candidates have been *NRAS*, *BRAF*, *PI3K* and phosphatase and tensin homologue (PTEN). Each of these will be dealt with in more detail in the next section.

1. *NRAS*

Mutations in *NRAS* are less common than *KRAS* and are present in approximately 2.6% of CRC cases [112]. As a result of this infrequency, only a limited number of studies investigating the predictive potential of *NRAS* have been undertaken. De Rooke et al. [112] showed a significant difference in response rate between patients in whom tumours showed an *NRAS* mutation (1/13, 7.7%) versus patients whose tumour was wild-type *NRAS* (110/289, 38.1%). Although, it should be noted that no significant difference in disease control rate, PFS or OS was observed.

2. BRAF

V-raf murine sarcoma viral oncogene homologue B1 (*BRAF*) is a serine-threonine kinase, which is downstream of RAS. *BRAF* is an important oncogene and is found to be mutated in a number of malignancies, including sporadic CRC, where the rate of mutation is of the order of 15% [108]. *BRAF* mutation has been associated with the serrated pathway of tumour development, and these tumours tend to be in groups 1 and 2 of the Jass classification [17]. The vast majority of *BRAF* mutations in CRC are the V600E mutation, which leads to constitutive activation of the kinase [122].

A number of the earlier studies examining biomarkers associated with a response to anti-EGFR therapy did not contain any CRC patients with a *BRAF* mutation. This has generally been a problem, where low patient numbers have led to poor statistical power. However, more recent research has overcome this, and studies have generally shown that the presence of a *BRAF* mutation correlates negatively with response to anti-EGFR therapy [98,112,123,124]. In a cohort of 173 CRC patients, Laurent-Puig et al. [123] noted that five patients with a *BRAF* mutation showed a significant decrease in PFS and OS when compared to *BRAF* wild-type patients treated with cetuximab. Although the numbers were small, this study did demonstrate statistical significance. De Roock et al. [112] have reported the largest cohort of *BRAF* mutant tumours to date with 36/761 (4.7%) of tumours tested containing a *BRAF* mutation. This group also demonstrated a significant difference in response rate between *BRAF* mutant and wild-type tumours.

Lin et al. [106] recently performed a systematic review of the predictive value of *BRAF* in mCRC. They concluded that, although the quality of the studies is impaired by their retrospective nature, there is definite evidence supporting the view that mutation of *BRAF* predicts a lack of response to anti-EGFR therapy. A subsequent meta-analysis by Mao et al. [125] has supported the results of these individual studies. This study observed that in *KRAS* wild-type patients, the objective response to anti-EGFR therapy was 0% in *BRAF* mutant tumours versus 36.3% in *BRAF* wild-type tumours. Further, Prahallad et al. [126] have recently suggested that BRAF (V600E) mutant colon cancers may benefit from a combined therapy of BRAF and EGFR inhibitors.

The data has now developed to the point where testing for *BRAF* is becoming part of the clinical routine for the management of mCRC patients in some centres. *BRAF* mutation testing is being incorporated into commercially available test kits for this purpose. It has also been shown that *KRAS* and *BRAF* analyses together have a favourable cost-benefit profile [119].

3. PIK3CA

Phosphatidylinositol-4,5-bisphosphate 3-kinase, catalytic subunit alpha, *PIK3CA*, is a phospholipid kinase, which is downstream of EGFR but signals via AKT rather than via the canonical MAP kinase pathway. As with *KRAS* and *BRAF*, *PIK3CA* undergoes activating mutations. The most common of these occurs in either exon 9 (approximately 60%, G1624A; E542K) or exon 20 (approximately 25%, A3140G; H1047R). These mutations result in different effects. Both are activating mutations, but exon 9 mutations require an interaction with activated RAS for subsequent downstream signalling. In the case of the exon 20 mutation, downstream signalling is independent of RAS [127].

There have been some suggestions that, in an *in vitro* setting at least, this may be the more important pathway in terms of the oncogenic effect of EGFR signalling [128]. Therefore, there has been significant interest in whether components of this pathway will impact on response to anti-EGFR therapy. As with *BRAF* studies, small cohort sizes have hampered attempts to definitively determine the predictive value of *PIK3CA* mutations.

There has been some conflicting clinical data regarding the importance of *PI3K* mutations in predicting response to anti-EGFR therapy. In their initial study, Lièvre et al. [104] did not show any correlation between *PIK3CA* mutation and response to cetuximab, but their study only included three patients with a *PIK3CA* mutation. A larger study (containing 15 tumours with a *PIK3CA* mutation) by Sartore-Bianchi et al. [129] showed a significant association between mutation of the gene and resistance to cetuximab or panitumumab. Similar results were obtained by Souglakos et al. [98]. However, these results were not replicated by others [130]. These differing results may have been reconciled by the work of De Roock et al. [112]. They [112] showed that exon 9 mutations had no effect on response to treatment, whilst exon 20 mutations were significantly correlated with objective response, PFS and OS. In their study, no patient with an exon 20 mutation responded to cetuximab-based therapy. A recent meta-analysis has supported these results, showing an objective response rate of 0% in patients whose tumours harboured a *PIK3CA* exon 20 mutation [125]. By contrast, the objective response rate was found to be 37% in patients with tumours that were wild-type for *PIK3CA* exon 20.

4. PTEN

PTEN is a key inhibitor of the *PIK* signalling pathway, and its expression has been shown to be lost in a number of different human tumours, including CRC. The mechanism of decreased expression can be driven by mutation or epigenetic phenomena, with up 13% of CRC in the COSMIC database showing a *PTEN* mutation. It has been suggested that this latter mechanism is particularly prevalent in tumours with high levels of MSI [131].

A number of CRC studies have examined PTEN expression and response to anti-EGFR therapy. These studies have primarily used IHC to assess expression at the protein level, and some have shown a correlation between PTEN expression and clinical response [123,132-134]. However, other studies have not shown such a relationship [135-137]. For example, Loupakis et al. [138] showed that PTEN expression in metastatic, but not in primary, tumours correlated with response to cetuximab. Moreover, Negri et al. [139] used indirect immunofluorescence to determine PTEN loss and showed that none of the patients with PTEN loss responded to cetuximab therapy. This was compared to a 30% disease progression rate in patients whose tumour retained PTEN expression [124]. These differing results may be due to the method of determining PTEN loss, i.e. IHC. Moreover, the studies examining PTEN have used differing protocols, antibodies and scoring systems, and this is reflected in the differing number of tumours found to be PTEN-deficient. This conflicting evidence has led the authors of at least one systematic review to conclude that, at present, PTEN expression levels cannot robustly predict response to anti-EGFR therapy [106]. Therefore, until there is further study in this area and until a standardised approach to assessment can be implemented, it appears that PTEN cannot be used in routine clinical practice.

5. pAKT

Closely associated with *PIK3CA* and PTEN testing, is activated AKT (pAKT). The presence of pAKT indicates activation of the pathway and therefore may indicate *PIK3CA* mutations, PTEN loss or activation of the pathway via a different mechanism. A number of studies have assessed the effect of pAKT expression in CRC and attempted to correlate it with response to anti-EGFR therapy. To date, however, no significant correlation has been found [138,140,141].

### Drug/diagnostic co-development—future approaches for personalised medicine

With the advance of our knowledge around patient segmentation, it will be necessary to consider the development of a companion diagnostic to aid treatment decision making during the drug development process. Ideally, the development of the diagnostic and the drug should happen in parallel, and the regulatory approval of both should be achieved simultaneously. Recent US FDA draft guidance on the development of companion diagnostics has been published [142]. In this draft guidance, the FDA sets forth its expectations for the development of companion diagnostic tools that will be used to determine treatment choices. This document also outlines the requirement for drug development and diagnostic tools to be carried out simultaneously. However, as recent events have indicated, this situation is not always achievable. In many circumstances, the understanding of the biology of the action of the drug in the intended disease population is evolving. Therefore, the knowledge of which biomarkers are important for the selection of appropriate patients for treatment may not be clear early enough in the programme to make the development of a diagnostic (in time for launch) a realistic goal. This is illustrated with the understanding around the role of *KRAS* mutations in resistance to anti-EGFR monoclonal antibody treatment. It is evident from recent clinical data from the phases II and III trials for panitumumab and cetuximab, where patients whose tumours harbour mutations in *KRAS* are less likely to respond to treatment. Retrospective analyses across seven randomised clinical trials suggest that anti-EGFR-directed monoclonal antibodies are not effective for the treatment of patients with mCRC containing *KRAS* mutations. This finding has led to the inclusion of information on the FDA-approved drug label for panitumumab and cetuximab (Indications and Usage section) to the effect that treatment of patients whose tumours harbour *KRAS* mutations is not recommended. Currently, however, there is no FDA-approved companion *KRAS* mutation test for either drug. The key rationale for *KRAS* mutation testing in this patient population is to avoid the exposure of patients to unnecessary drug toxicities in situations where there is unlikely to be any clinical benefit. In addition, this avoids unnecessary financial burdens on the healthcare system. However, it does remain that CRC patients whose tumours harbour *KRAS* mutations have a high unmet medical need. Understanding the role of additional biomarkers in CRC and their potential use as predictive markers of drug response will show possible utility for the future and will allow us to see an increase in the development of companion diagnostic tools to support this.

### Recent National Institute for Health and Clinical Excellence ruling on use of bevacizumab

The UK National Institute for Health and Clinical Excellence has recently published guidelines on the use of VEGF- and EGFR-targeted therapies in treating mCRC patients who have failed to respond adequately to first-line treatments [143]. The guidelines have acknowledged the fact that cetuximab showed benefits in terms of PFS and OS when used in KRAS wild-type mCRC patients compared with BSC alone. They also noted that panitumumab, given as a monotherapy, had shown a benefit in terms of PFS. However, following cost-benefit analysis, the committee concluded that cetuximab and panitumumab (in addition to bevacizumab) were ‘not a cost-effective use of National Health Service (NHS) resources’ [143]. This analysis highlights the need to continue to refine our strategies in terms of the use of biomarkers and to identify those patients who are likely to derive the most benefit from these treatments. This will not only promote genuine personalised medicine, but will also enable us to use these therapies in a manner that will not put undue strain on finite healthcare resources.

## Conclusions

In recent years, significant steps have been made in the diagnosis and successful treatment of CRC. Researchers and clinicians, however, are still faced with challenges, not least the detection and management of tumours with varied gene mutation status. Further clarification of the molecular pathology of CRC may improve treatment options as well as improve QoL and ultimately, the long-term survival of patients with this condition. As new technologies emerge allowing the identification of CRC gene mutation status, this information will inform clinicians as to the most appropriate and efficacious form of treatment. This individualised approach to managing mCRC fits in well with the PPPM model put forward by EPMA [144,145]. However, in order to gain most benefit from these novel technologies, targeted therapy must be informed by well-designed studies, large cohort sizes and internationally standardised detection protocols. Therefore, it is imperative that scientists and clinicians collaborate closely in developing new ways to deal with the vast quantities of data generated by mutational analyses in order to make sense of the underlying mechanisms of CRC. As human populations are living longer, healthcare systems around the world will be challenged to find new ways of dealing with CRC to improve global health.

### Expert recommendations

The following are expert recommendations:

• Routine *KRAS* and *BRAF* mutation testing of patients with mCRC who are being considered for anti-EGFR therapy to ensure they receive the most appropriate therapy whilst being spared exposure to non-beneficial therapeutic effects.

• *PIK3CA* and PTEN have also shown promise as informative biomarkers for EGFR therapy, but further studies are necessary before they can be used clinically on a routine basis.

• Panel-based biomarker testing platforms should be developed as a way to ensure that maximal value can be derived from limited clinical material.

• VEGF inhibitors have shown promise in the treatment of mCRC, but more research is required into identifying predictive markers for patient response. This will allow a more personalised approach to anti-VEGF treatments in the future.

• Access to high-quality tumour material for biomarker testing is of paramount importance; therefore, tissue collection and storage procedures must be standardised internationally.

• Development of internationally accessible bio-banks for CRC tissues is necessary to progress high-quality biomarker research.

• Good practice is essential in maintaining detailed patient records for developing PPPM, based on multifactorial data, e.g. sex, age, weight, ethnicity, lifestyle, and prior therapy regimens.

• High stringency of genomic and proteomic assays is required across research labs to ensure reproducibility of data.

• Early biomarker discovery, coupled with approaches to ensure appropriate co-development of formative companion diagnostics, should be a standard practice in all drug development programmes.

• Improved clinical trial design with more stringent stratification of patient cohorts.

## Abbreviations

5-FU: 5-fluorouracil; BSC: Best supportive care; CIMP: CpG island methylator phenotype; CIS: Chromosomal instability; CRC: Colorectal cancer; DCE-MRI: Dynamic contrast enhanced-magnetic resonance imaging; EGFR: Epidermal growth factor receptor; FDA: Food and Drugs Administration; FLT-PET: ^18^F-fluorothymidine-positron emission tomography; HNPCC: Hereditary non-polyposis colorectal cancer; IHC: Immunohistochemistry; mCRC: Metastatic colorectal cancer; MMR: Mismatch repair; MSI: Microsatellite instability; MSI-H: High level microsatellite instability; MSI-L: Low level microsatellite instability; MSS: microsatellite stable; NHS: National Health Service (UK); OS: Overall survival; PFS: Progression-free survival; *PI3K*: Phosphatidylinositol 3-kinase; PlGF: Placental growth factor; PPPM: Predictive, preventive and personalised medicine; PTEN: Phosphatase and tensin homologue; QoL: Quality of life; sVEGFR-1: Soluble VEGF receptor 1; TKI: Tyrosine kinase inhibitors; VEGF: Vascular endothelial growth factor.

## Competing interests

The authors declare that they have no competing interests.

## Authors’ contributions

SH and BD conceived the review, and SH coordinated the drafting of the manuscript. SH, MO and BD participated in the design of the review, performed literature searches and identified relevant studies. BD and MO provided content expertise. All authors read and approved the final manuscript.

## Authors’ information

SH (PhD FEA) is a lecturer in Anatomy and Physiology in the Department of Life Sciences at GCU, with a special interest in predictive and prognostic biomarkers of inflammation and disease. Her previous research background included publications on the metastasis suppressor gene, RKIP, in mCRC and breast cancer. MO is a Diagnostic Team Director in the Personalised Healthcare and Biomarker Function within AstraZeneca, where her role involves her in championing the use of biomarkers to support oncology drug development. BD (MB BAO BCh PhD FRCPath) is a lecturer in Histopathology at Trinity College Dublin, with a special interest in gastrointestinal and molecular pathology.
